# Risk Factors for Healthcare-Associated Infections After Pediatric Cardiac Surgery*

**DOI:** 10.1097/PCC.0000000000001445

**Published:** 2018-03-02

**Authors:** Takeshi Hatachi, Kazuya Tachibana, Yu Inata, Yuji Tominaga, Aiko Hirano, Miyako Kyogoku, Kazue Moon, Yoshiyuki Shimizu, Kanako Isaka, Muneyuki Takeuchi

**Affiliations:** 1 Department of Intensive Care Medicine, Osaka Women’s and Children’s Hospital, Osaka, Japan.; 2 Department of Anesthesiology, Osaka Women’s and Children’s Hospital, Osaka, Japan.; 3 Department of Cardiovascular Surgery, Osaka Women’s and Children’s Hospital, Osaka, Japan.

**Keywords:** cardiac surgery, dopamine, healthcare-associated infections, intensive care units, pediatrics

## Abstract

**Objectives:**

Healthcare-associated infections after pediatric cardiac surgery are significant causes of morbidity and mortality. We aimed to identify the risk factors for the occurrence of healthcare-associated infections after pediatric cardiac surgery.

**Design:**

Retrospective, single-center observational study.

**Setting:**

PICU at a tertiary children’s hospital.

**Patients:**

Consecutive pediatric patients less than or equal to 18 years old admitted to the PICU after cardiac surgery, between January 2013 and December 2015.

**Interventions:**

None.

**Measurements and Main Results:**

All the data were retrospectively collected from the medical records of patients. We assessed the first surgery during a single PICU stay and identified four common healthcare-associated infections, including bloodstream infection, surgical site infection, pneumonia, and urinary tract infection, according to the definitions of the Centers for Disease Control and Prevention and National Healthcare Safety Network. We assessed the pre-, intra-, and early postoperative potential risk factors for these healthcare-associated infections via multivariable analysis. In total, 526 cardiac surgeries (394 patients) were included. We identified 81 cases of healthcare-associated infections, including, bloodstream infections (*n* = 30), surgical site infections (*n* = 30), urinary tract infections (*n* = 13), and pneumonia (*n* = 8). In the case of 71 of the surgeries (13.5%), at least one healthcare-associated infection was reported. Multivariable analysis indicated the following risk factors for postoperative healthcare-associated infections: mechanical ventilation greater than or equal to 3 days (odds ratio, 4.81; 95% CI, 1.89–12.8), dopamine use (odds ratio, 3.87; 95% CI, 1.53–10.3), genetic abnormality (odds ratio, 2.53; 95% CI, 1.17–5.45), and delayed sternal closure (odds ratio, 3.78; 95% CI, 1.16–12.8).

**Conclusions:**

Mechanical ventilation greater than or equal to 3 days, dopamine use, genetic abnormality, and delayed sternal closure were associated with healthcare-associated infections after pediatric cardiac surgery. Since the use of dopamine is an easily modifiable risk factor, and may serve as a potential target to reduce healthcare-associated infections, further studies are needed to establish whether dopamine negatively impacts the development of healthcare-associated infections.

In the United States of America and Japan, approximately 40,000 and 9,000 children, respectively, undergo pediatric cardiac surgery, annually, and the overall mortalities are 3.1% and 2.3%, respectively; these values have significantly improved in recent decades (1, 2). However, postoperative healthcare-associated infections (HAIs) after pediatric cardiac surgery remain significant causes of morbidity and mortality (3–8).

Despite the wide acceptance of guidelines and prevention bundles, the frequency of HAIs remains high, at 6.0–30.8%, after pediatric cardiac surgery (3, 5, 6, 9). Therefore, it is essential to identify and modify the risk factors for postoperative HAIs.

Several studies have reported various risk factors for postoperative HAIs (3–5, 9–11). However, none of those studies evaluated the risk factors throughout the perioperative period for consecutive pediatric patients and for all four common HAIs, including bloodstream infection (BSI), surgical site infection (SSI), pneumonia, and urinary tract infection (UTI) (6, 9). Furthermore, dopamine has never been assessed as a potential risk factor for HAIs after pediatric cardiac surgery despite its association with HAIs in children with sepsis (12). Therefore, we included dopamine and other inotropes as potential risk factors for postoperative HAIs.

In the present study, we aimed to identify pre-, intra-, and early postoperative risk factors for these four common HAIs after pediatric cardiac surgery, including the use of dopamine and other inotropes.

## MATERIALS AND METHODS

### Design and Setting

We conducted a retrospective observational study of pediatric patients admitted to the PICU at the Osaka Women’s and Children’s Hospital in Osaka, Japan, after cardiac surgery, from January 1, 2013, to December 31, 2015. The PICU had 8–12 beds, during the study period, for medical and surgical pediatric patients. Approximately 400 pediatric patients were admitted to the PICU annually, with approximately 190 of these patients being admitted after cardiac surgery. Heart transplantation was not performed at our hospital.

This study was approved by the hospital’s Ethics Committee, and the need for informed consent was waived due to its retrospective nature.

### Inclusion and Exclusion Criteria

We included all consecutive cases of pediatric cardiac surgery performed in patients less than or equal to 18 years old admitted to the PICU. We excluded surgical procedures if they were performed in patients in the neonatal ICU. We excluded patients who underwent pacemaker implantation, aortopexy, tracheoinnominate artery fistula ligation, tumor excision, and cardiac surgery after noncardiac thoracic surgery during the same PICU stay. We excluded patients who died within 48 hours following surgery.

### Clinical Endpoint

The primary endpoint was the occurrence of an HAI, such as BSI, SSI, pneumonia, and UTI, after pediatric cardiac surgery.

### Definitions of HAIs

Postoperative HAIs were defined as cases of HAI diagnosed after surgery, with no evidence that the infection was present or incubating at the time of surgery. In the present study, BSI, SSI, pneumonia, and UTI were retrospectively diagnosed, according to the 2008 definitions of the Centers for Disease Control and Prevention and National Healthcare Safety Network (CDC/NHSN) (13). All the patients were surveyed up to 48 hours after discharge from the PICU. With regard to SSI, patients were surveyed up to 30 days for superficial incisional SSI and up to 90 days for deep incisional SSI and organ/space SSI, after surgery. The HAIs were diagnosed independently by two doctors.

BSI included both laboratory-confirmed BSI (LCBSI) and clinical sepsis. LCBSI was defined when a pathogen, not related to another site, was cultured from greater than or equal to one blood cultures. In addition, when a pathogen was a common skin contaminant (e.g., coagulase-negative staphylococci), greater than or equal to two positive cultures were needed to define LCBSI. Clinical sepsis was defined when a patient less than or equal to 1 year old exhibited signs or symptoms of infection; a physician then instituted treatment for sepsis where the blood culture test was either not performed or indicated negative results.

SSIs included incisional SSI and organ/space SSI. Incisional SSI consisted of superficial SSI, which involved only the skin and subcutaneous tissue, and deep incisional SSI, which involved the deep soft tissues. Organ/space SSIs involved organs or spaces that were exposed during surgery.

Pneumonia was defined as cases in which 1) infiltrate or consolidation was observed on a chest radiograph; 2) signs or symptoms (fever, leukopenia, or leukocytosis) were detected; and 3) worsening sputum or respiratory function was observed.

UTI was confirmed when a patient had at least one of the signs or symptoms and one of the test results shown below.

Signs or symptoms: Fever (body temperature > 38°C), urgency, frequency, dysuria, suprapubic tenderness, or costovertebral angle pain or tenderness.

Test results are as follows:

1) A positive urine culture of greater than or equal to 10^5^ colony-forming units per mL.
2) At least one of the following: 1) positive dipstick for leukocyte esterase and/or nitrite, 2) pyuria, or 3) microorganisms observed on Gram stain.

Catheter-associated UTI was defined as UTI in the presence of a urinary catheter, within 48 hours.

### Prevention and Control Strategies for HAIs

Patients received prophylactic antibiotic therapy with cefazolin (60 mg/kg a day), which was initiated during the induction of anesthesia and continued until 48 hours following surgery. Patients proven to have nasal colonization of methicillin-resistant *Staphylococcus aureus* (MRSA) received intranasal mupirocin for 3 days before surgery, prophylactic vancomycin (15 mg/kg per dose) during the operative period, and cefmetazole (60 mg/kg a day) until 48 hours after surgery. Patients who experienced delayed sternal closure (DSC) from January 2013 to December 2014 received prophylactic vancomycin (15 mg/kg per dose) and meropenem (60 mg/kg a day) until 48 hours of sternal closure, whereas those with this condition from January 2015 onwards received only cefazolin (60 mg/kg a day) until 48 hours of sternal closure. Preoperative disinfection of the skin was performed with 70% alcohol, followed by povidone iodine. A single dose of methylprednisolone (30 mg/kg) was administered to all patients who underwent cardiopulmonary bypass (CPB). The insertion of and maintenance bundles for the central venous catheter and urinary catheter and a ventilator-associated pneumonia prevention program were conducted based on the CDC/NHSN guidelines. Institutional guidelines were provided, and healthcare personnel were educated about the indication, insertion, and maintenance of the devices. For BSI prevention, trained personnel inserted central venous catheters using aseptic techniques and maximum sterile barrier precautions. For skin preparation, povidone iodine was used as alcoholic chlorhexidine antiseptic was not available. For UTI prevention, a closed drainage system for urinary catheters was implemented. For ventilator-associated pneumonia prevention, the readiness to extubate was assessed daily. Oral care was provided, and the head of the patient was elevated. We did not use a prospective surveillance program, to monitor the adherence to the guideline.

### Data Collection

All the data were retrospectively collected from the medical records of patients. If a patient had undergone greater than or equal to two surgeries during a single PICU stay, only the first surgery was assessed. We recorded the number of surgeries, number of patients, and patients’ demographic data. We defined surgical complexity using the Risk Adjustment for Congenital Heart Surgery (RACHS)–1 category (14). Each RACHS-1 category included cardiac surgeries that had a similar mortality, with group 1 having the lowest risk of mortality and group 6 having the highest risk. We calculated the number of patients who underwent CPB during the surgical procedure, length of mechanical ventilation, length of postoperative PICU stay, length of postoperative hospital stay, and mortality up to 28 days after discharge from the PICU. We also identified the number of preoperative and postoperative HAIs. In addition, we identified the HAI sites, pathogens, and the day of diagnosis postoperatively.

### Potential Risk Factors for Postoperative HAIs

The screened potential risk factors for postoperative HAIs, based on the literature and our consideration, were as follows.

#### Preoperative.

The preoperative potential risk factors included age less than 6 months (3–6, 9–11), sex (3–5, 10), genetic abnormality (3–5, 10), absence of splenic function (10), preoperative PICU stay (3, 4, 9, 10), and nasal colonization of MRSA (15, 16). Race was not included because Japan is almost a racially homogeneous nation.

#### Intraoperative.

The intraoperative potential risk factors included RACHS-1 score greater than or equal to 3 (4, 5, 9, 11), American Society of Anesthesiologists score greater than or equal to 3 (10), surgery duration greater than 3 hours (3, 9, 10), CPB (3–5, 9, 10), and lowest core temperature during surgery less than 32°C (3, 9, 10).

#### Postoperative.

The postoperative potential risk factors included DSC (3, 5, 6, 9–11), extracorporeal membrane oxygenation (ECMO) (3, 5), mechanical ventilation for greater than or equal to 3 days (3, 5, 6, 9, 10), PICU stay for greater than or equal to 3 days (6, 9, 11), peritoneal dialysis or continuous hemodiafiltration (PD/CHDF), RBC transfusion within 2 days of surgery (3, 9), use of postoperative steroids (3, 10), peak glucose greater than 360 mg/dL within 2 days of surgery (9, 10), multiple surgeries during a single PICU stay (4), and the use of dopamine (12) and other inotropes including epinephrine, norepinephrine, and dobutamine within 3 days of surgery. ECMO and PD/CHDF were considered when they were performed within 3 days of surgery. Postoperative steroids, including hydrocortisone, prednisolone, and dexamethasone, were considered when they were administered within 3 days of surgery. Multiple surgeries during a single PICU stay were defined when greater than or equal to two separate cardiovascular surgeries were performed during the same PICU stay, prior to the development of HAIs; these did not include surgery to stop bleeding or surgery for sternal closure.

### Effects of Dopamine

To examine the effects of dopamine on the frequency of postoperative HAIs, we stratified patients into two groups: with dopamine and without dopamine, in which all the other risk factors (except for dopamine use) were adjusted through propensity score matching. Then, we compared the frequency of postoperative HAIs, with and without dopamine.

In addition, we examined the dose-dependent effect of dopamine on postoperative HAIs. First, we stratified patients into three groups: patients who did not receive dopamine, patients who received dopamine for 3 days or less, and patients who received dopamine for more than 3 days. Then, we compared the frequency of postoperative HAIs among these groups. Second, we stratified patients depending on the total amount of dopamine (mg) used per kg of body weight within 3 days of surgery, into four groups: total amount of dopamine ≤ 5 mg/kg, > 5 and ≤ 10 mg/kg, > 10 and ≤ 15 mg/kg, and > 15 mg/kg. We compared the frequency of postoperative HAIs among these groups. Furthermore, to differentiate the dopamine effect from the inotrope effect, we added a maximum Vasoactive-Inotrope Score (17, 18) during the first 3 days following surgery into the additional multivariable analysis, for postoperative HAIs.

### Statistical Methods

Categorical variables were evaluated using the chi-square test or Fisher exact test, as appropriate. Continuous variables were evaluated using the Wilcoxon rank-sum test. Statistical significance was defined as *p* value of less than or equal to 0.05. The individual potential risk factors for HAIs from the literature and our consideration were assessed via bivariate analysis. The significant risk factors (*p* < 0.1) in the bivariate analysis were included in multivariable logistic regression analysis. The risk factors highly correlated in the bivariate analysis (Cramer’s V > 0.7) were not included in the multivariable analysis together. Propensity score matching method was used to adjust the risk factors for dopamine use. The statistical analyses were conducted using JMP Version 10.0 (SAS Institute, Cary, NC) and Stata Version 14.0 (Stata Corp LLC, College Station, TX).

## RESULTS

### Study Surgical Procedures and Population

During the study period, 571 cardiac surgeries were performed, and 526 surgeries (394 patients) were eligible for inclusion. A total of 45 surgeries were excluded, such as those performed on patients greater than 18 years old (*n* = 6) and those performed in the neonatal ICU (*n* = 6). Patients undergoing pacemaker implantation (*n* = 11), aortopexy (*n* = 1), tracheoinnominate artery fistula ligation (*n* = 1), and cardiac tumor excision (*n* = 1), as well as those with a history of repair of esophageal atresia (*n* = 1) were also excluded. Eleven, two, and one patient/s underwent two, three, and four surgeries, respectively, during a single PICU stay. We assessed only the first surgery during a single PICU stay, and hence, the second, third, and fourth surgeries were excluded (*n* = 18). None of the patients died within 48 hours following surgery. The number of patients in each RACHS-1 score category was as follows: 57 (11%) in category 1, 175 (33%) in category 2, 238 (45%) in category 3, 31 (6%) in category 4, none in category 5, and 25 (5%) in category 6. A total of 76.6% surgeries were performed with the patient under CPB (*n* = 403). **Table 1** shows the patient characteristics and clinical outcomes, based on the presence of HAIs.

**TABLE 1.**
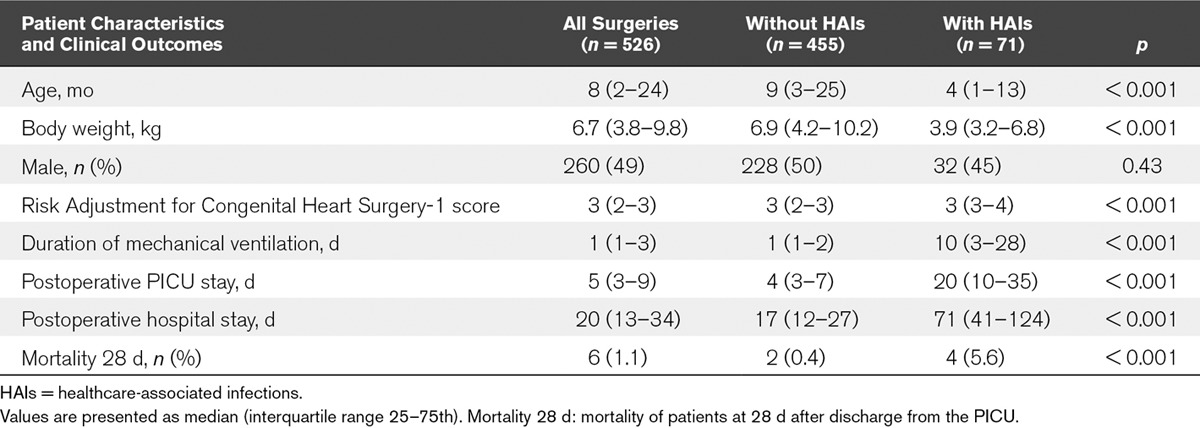
Patient Characteristics and Clinical Outcomes Based on the Presence of Healthcare-Associated Infections

### HAIs and Causative Pathogens

In the cases of 526 surgeries, 81 postoperative HAIs were identified. **Table 2** shows the number of postoperative HAIs, causative pathogens, and postoperative days on which HAIs were diagnosed. Of all the eligible surgeries, one surgery led to three postoperative HAIs during a single PICU stay, whereas eight surgeries led to two postoperative HAIs. Therefore, 71 surgeries had at least one postoperative HAI. The overall frequency of postoperative HAIs was 13.5 per 100 surgeries.

**TABLE 2.**
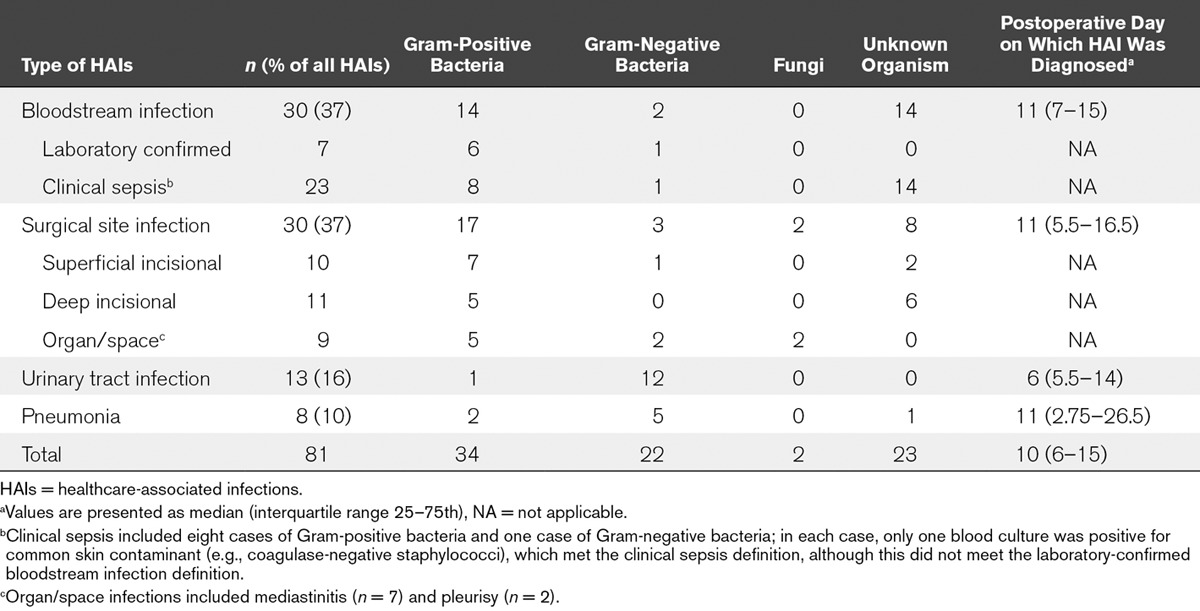
Number of Postoperative Healthcare-Associated Infections, Causative Pathogens, and Postoperative Day on Which the Healthcare-Associated Infection Was Diagnosed

Seven HAIs, including UTIs (*n* = 4), BSIs (*n* = 2), and pneumonia (*n* = 1), presented before the surgical procedure. Two patients had both preoperative and postoperative HAIs; however, the causative pathogens and sites of infection were not the same between the preoperative and postoperative HAIs.

### Risk Factors for Postoperative HAIs

**Table 3** shows the potential risk factors for postoperative HAIs in the bivariate analysis. The potential risk factors that were significant in the bivariate analysis (*p* < 0.1) were assessed for their correlation with each other, and none had a high correlation (Cramer’s V > 0.7) with another. Therefore, all the potential risk factors that were significant in the bivariate analysis were included in the multivariable analysis. The potential risk factors for postoperative HAIs included in the final multivariate analysis are shown in Table 3. **Table 4** shows the risk factors for postoperative HAIs in the multivariable analysis. The significant risk factors for postoperative HAIs included mechanical ventilation greater than or equal to 3 days, dopamine use, genetic abnormality, and DSC.

**TABLE 3.**
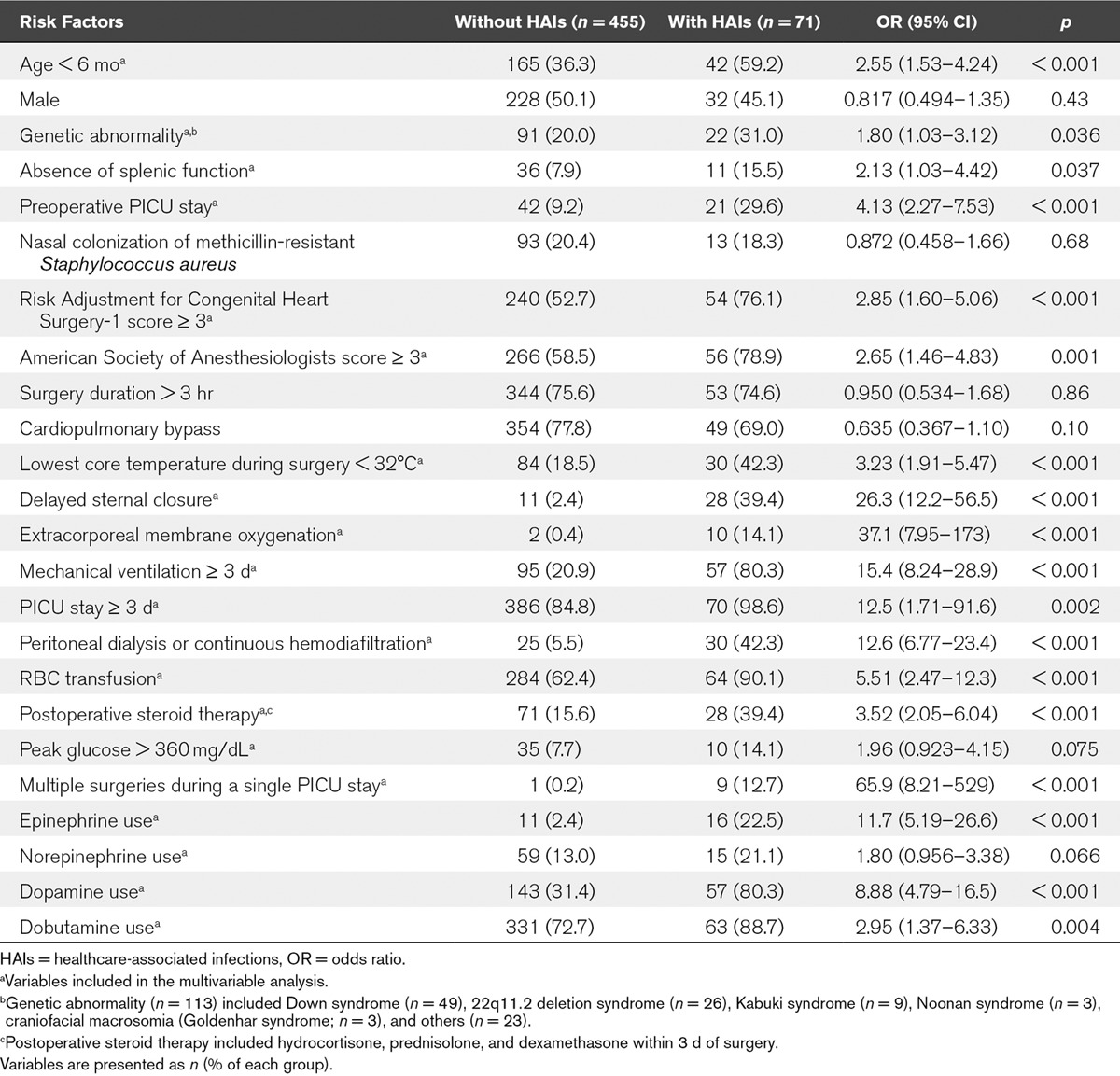
Potential Risk Factors for Postoperative Healthcare-Associated Infections in Bivariate Analysis

**TABLE 4.**
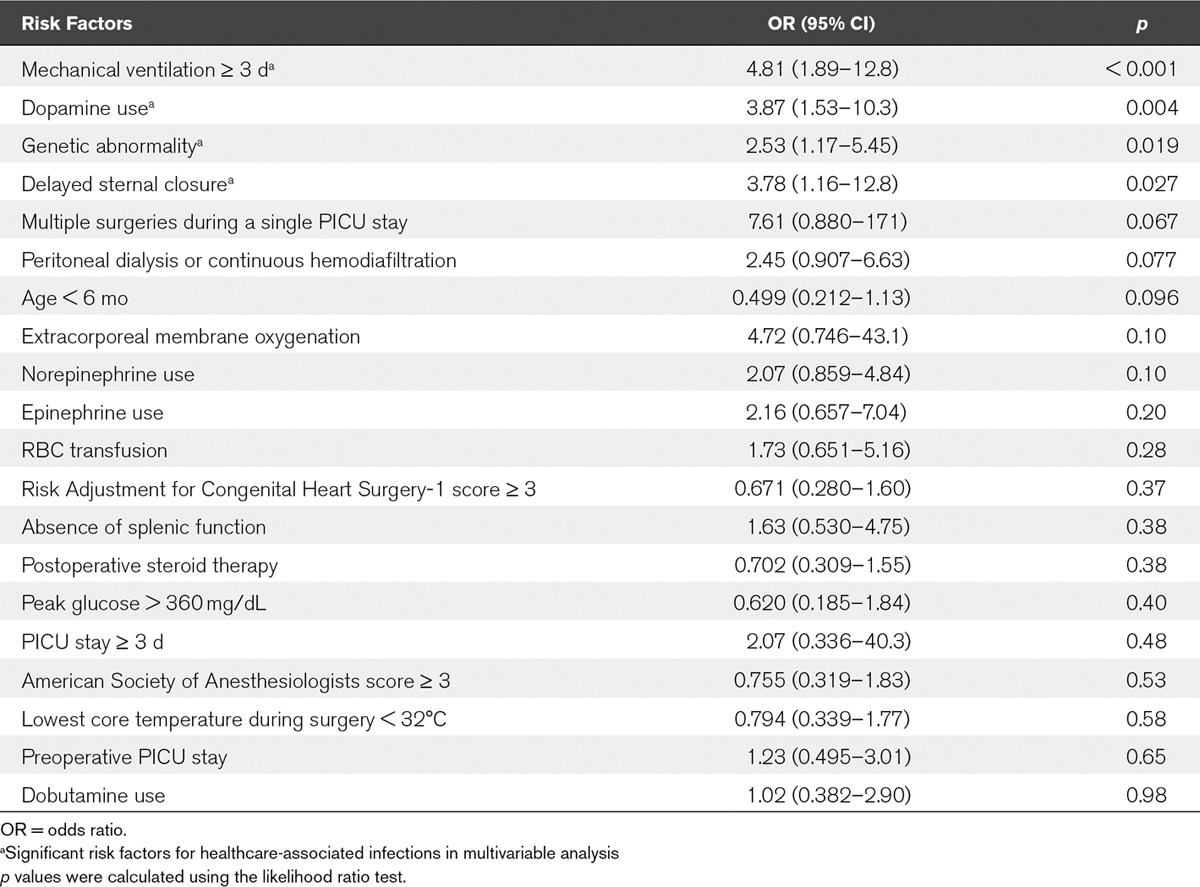
Risk Factors for Postoperative Healthcare-Associated Infections in Multivariable Analysis

### Effects of Dopamine

After adjusting the 23 potential risk factors included in Table 3 (except for the use of dopamine), through propensity score matching, 200 patients with dopamine and 200 controls without dopamine were compared, with regard to the occurrence rates of HAIs. The frequencies of postoperative HAIs, among patients with and without dopamine, were 57 of 200 (29%) and 12 of 200 (6%) (odds ratio, 6.24; 95% CI; 3.23–12.1; *p* < 0.001).

With regard to the dose-dependent effect, the frequencies of postoperative HAIs were 4.3% (14/326), 16.3% (13/80), and 36.7% (44/120), respectively, among patients who did not receive dopamine, patients who received dopamine for 3 days or less, and patients who received dopamine for more than 3 days (*p* < 0.001). In addition, the frequencies of postoperative HAIs were 15% (5/34), 17% (9/54), 38% (20/52), and 38% (23/60), respectively, among patients with a total amount of dopamine ≤ 5 mg/kg, > 5 and ≤ 10 mg/kg, > 10 and ≤ 15 mg/kg, and > 15 mg/kg (*p* = 0.007).

With regard to the vasoactive inotropic effect, in the additional multivariable analysis which included a maximum Vasoactive-Inotrope Score during the first 3 days of surgery, dopamine use was a risk factor for postoperative HAIs (*p*= 0.005), whereas the use of inotrope was not (*p* = 0.76).

## DISCUSSION

In this retrospective study, we demonstrated that mechanical ventilation greater than or equal to 3 days, dopamine use, genetic abnormality, and DSC were independently associated with the four common postoperative HAIs. To the best of our knowledge, this is the first study to examine the potential risk factors during the pre-, intra-, and postoperative periods, for these common HAIs, and to demonstrate the association between dopamine use and frequencies of HAI after pediatric cardiac surgery.

The role of dopamine in critically ill patients has been questioned in various clinical settings (19, 20). Dopamine can modulate immune responses and may result in the inhibition of cytokine and chemokine production, inhibition of neutrophil chemotaxis, and disturbance of T-cell proliferation (21). In animal studies, bromocriptine—a dopamine receptor agonist that inhibits pituitary prolactin secretion—was found to suppress T-lymphocyte–dependent macrophage activation (22); similarly, it was also found to suppress the activity of T lymphocytes (23). Furthermore, dopamine suppressed prolactin levels and the T-cell response, in human studies (24, 25). Despite these theoretical disadvantages of immunosuppression, dopamine is still widely used in various clinical settings, including pediatric cardiac surgery (26–29). Recently, Ventura et al (12) compared the use of dopamine and epinephrine for pediatric septic shock in a randomized controlled trial and found that the rate of HAIs was higher in patients treated with dopamine than in those treated with epinephrine. However, no studies have investigated the association between dopamine use and postoperative HAIs in pediatric patients after cardiac surgery. In the present study, we identified dopamine use as an independent risk factor for HAIs after pediatric cardiac surgery; in addition, this factor is modifiable.

Dopamine was administered at the discretion of the treating providers; therefore, we adjusted for the factors associated with the use of dopamine through propensity score matching. Significant differences in the frequencies of postoperative HAIs between patients with and without dopamine remained, after adjusting the patient-level differences associated with dopamine use. In addition, we examined the dose-dependent effect of dopamine on postoperative HAIs. The duration of dopamine administration and the total amount of dopamine per kg of body weight were associated with postoperative HAIs in the bivariate analysis, and the presence of dose-dependent effects of dopamine on postoperative HAIs was noted. Furthermore, to differentiate the dopamine and inotrope effects, we added a maximum Vasoactive-Inotrope Score and found that while dopamine use remained a risk factor for postoperative HAIs, the use of inotrope did not.

The duration of mechanical ventilation, genetic abnormality, and DSC were reported as risk factors for postoperative HAIs in other studies, although they are not easily modifiable. In particular, mechanical ventilation was found to be a risk factor for postoperative HAIs (3), pneumonia (30), and BSI (31), after pediatric cardiac surgery. Invasive tracheal intubation leads to airway damage, and the tracheal tube serves as a reservoir for bacteria (32). The use of noninvasive ventilation, protocolized sedation, and a respiratory care program reduced the frequency of frequencies of postoperative HAIs (33, 34). Genetic abnormality is also a risk factor for postoperative HAIs (4, 5). The frequency of SSI was higher in children with 22q11.2 deletion syndrome (35), who were reported to have immunodeficiency (36). Although DSC has hemodynamic and respiratory benefits (37), it is a risk factor for postoperative HAIs (5, 9, 11), and prolonged DSC may increase the frequency of HAIs (38). Hence, minimizing the duration of DSC should be considered.

The present study had certain limitations. First, it was conducted in a single center, which limits the generalizability of the results. Furthermore, the number of patients may not be sufficiently large to identify the other risk factors for HAIs. HAIs were detected in 13.5% of the cases of surgeries, which is similar to the data obtained from other studies (6.0–30.8%) (3, 5, 6, 9). The most common HAIs included BSIs and SSIs, and the common causative pathogens were Gram-positive bacteria for BSIs (14/30) and SSIs (17/30), and Gram-negative bacteria for UTIs (12/13) and pneumonia (5/8), as was noted in other studies (3, 6, 9, 10). Furthermore, our study population, the frequency and type of HAIs, and the causative pathogens were similar to those observed in other studies, reflecting the generalizability of our study. Second, the study was retrospective in nature. Hence, infection prevention and control strategies could not be manipulated. In our study, of the patients with nasal MRSA colonization, the frequencies of postoperative HAIs in the patients who received prophylactic vancomycin and those who did not were 16% and 3% (*p* = 0.06), respectively. In addition, the prophylactic antibiotic regimen for DSC was changed during the study period, making it difficult to analyze infections during the two-time frames, together. Nonetheless, there was no statistically significant difference in the frequency of HAIs between patients with DSC (65% during the vancomycin plus meropenem period and 79% during the cefazolin period) (*p* = 0.33). Similarly, the strategies for inotrope use, RBC transfusion, and postoperative steroid use could not be controlled and were based on the physician’s clinical decision. It is possible that the type of surgery affected the use of dopamine, as well as the frequency of postoperative HAIs. Although there are no documented guidelines for inotrope use, dobutamine was the most commonly used inotrope for postoperative low cardiac output syndrome, and milrinone was added when elevated systemic vascular resistance was observed. Dopamine was used initially, and epinephrine was the next choice when low systemic vascular resistance was observed. In addition, all the diagnoses of HAIs were retrospective. There was a possibility that the severity of illness affected the use of therapeutic antibiotics and the diagnosis of postoperative HAIs. Furthermore, some HAIs, especially cases of clinical sepsis which are diagnosed according to clinical manifestations, may have been overlooked. Nonetheless, during diagnosis, we prospectively reviewed the recorded documents on suspected infections, including the results of cultures, symptoms (i.e., fever, hypotension, and apnea), use of antibiotics, and results of blood tests. Third, we assessed only the first surgeries during a single PICU stay. Nevertheless, we included multiple surgeries during a single PICU stay as potential risk factors for postoperative HAIs. Interestingly, undergoing multiple surgeries during a single PICU stay was not an independent risk factor for HAIs. Fourth, although we employed the 2008 CDC/NHSN surveillance definition (13), according to the latest definition, only LCBSI is considered a BSI and is grouped into central line–associated BSI (CLABSI) and non-CLABSI. Therefore, we may have overdiagnosed BSI by including cases without positive blood cultures; however, we believe that those episodes may have been clinically important. Fifth, the U.S. guideline recommends the use of alcohol-containing preoperative skin preparatory agents (39), which were not available in the study period in Japan; therefore, we used 70% alcohol followed by povidone iodine for preoperative skin antisepsis.

## CONCLUSIONS

This retrospective, single-center study identified the risk factors for BSI, SSI, pneumonia, and UTI, following pediatric cardiac surgery. The frequency of HAIs after pediatric cardiac surgery was 13.5%. The risk factors for HAIs after pediatric cardiac surgery include mechanical ventilation greater than or equal to 3 days, dopamine use, genetic abnormality, and DSC. Since the use of dopamine is an easily modifiable risk factor, and may serve as a potential target to reduce HAIs, further studies are needed to establish whether dopamine negatively impacts the development of HAIs.

## ACKNOWLEDGMENTS

We thank the staff at our PICU for cooperating with us on this study, particularly Masayo Tsuda, Hideyuki Matsunaga, Nao Okuda, Takaaki Akamatsu, Noboru Matsumoto, and Atsushi Kawamura (PICU physicians). We would also like to thank Kenji Hirai (health information manager), Akihito Inoue (medical engineer), Makie Kinoshita (infection control nurse), and Futoshi Fujiwara (clinical microbiology laboratory personnel) for their assistance with data collection, as well as Kimiko Ueda and Yusuke Naito for their assistance with the statistical analyses.
